# Development of a Novel Piezoelectric Harvester Excited by Raindrops

**DOI:** 10.3390/s19173653

**Published:** 2019-08-22

**Authors:** Alberto Doria, Giulio Fanti, Gino Filipi, Federico Moro

**Affiliations:** Department of Industrial Engineering, University of Padova, 35131 Padova, Italy

**Keywords:** raindrop energy harvester, impulse vibrations, piezoelectric

## Abstract

The impact of raindrops on a dry surface leads to a splashing phenomenon that dissipates a lot of energy. To improve energy collection, a novel piezoelectric raindrop energy harvester equipped with a spoonful of water was developed. The advantages and the drawbacks of this solution were analyzed with the aid of numerical simulations. A series of experimental tests were carried out in a laboratory with simulated raindrops. Experimental results showed that the negative effect of the added water mass was exceeded by the positive effects related to the impact of the raindrop on a liquid surface. Tests carried out connecting the harvester to a resistive load showed that the prototype was able to collect more energy than a simple cantilever harvester.

## 1. Introduction

On sunny days, relevant amounts of energy can be harvested by means of solar panels. This technology is mature and nowadays it is widely used both for generating electric power, which can be injected into the grid, and for feeding small end users, which are not connected to the grid. These small users include buildings in remote areas, sensors, antennas, and monitoring equipment. On rainy days, nature offers a different kind of energy. Raindrops impact the ground with a kinetic energy that usually is lost, but that can be scavenged by means of piezoelectric harvesters [1,2,3].

In temperate climate countries, energy harvested from raindrops is enough to feed small equipment like sensors with RF transmitters, since the power consumption of modern electronic devices is constantly decreasing [4]. In tropical countries with large rain rates, the energy harvested from raindrops is larger and, in the future, it could be used for feeding small electric/electronic appliances in remote areas [5,6].

After the pioneering theoretical and experimental studies of Guigon et al. in 2008 [1,2], the research on raindrop harvesters have been focused on several different topics. Some researchers have studied the shape, material, and circuitry of piezoelectric devices. Different layouts of the system were proposed and tested by means of prototypes [7,8,9], most of them were obtained by combing in different ways piezoelectric cantilevers, this structure is still the reference in the field of raindrop harvesters. Tests reported in [3] showed that, if the structure is very thin (some tens of μm), a bridge configuration generates higher open-circuit voltage than a cantilever configuration, whereas results reported in [7] showed that if the harvester structure is thicker, the cantilever configuration generates higher open-circuit voltage than the bridge or floating circle configuration. The effect of piezoelectric materials on the performance of the system was studied in [3,10]. PZT (lead zirconate titanate) and PVDF (polyvinylidene fluoride) are nowadays the most widely used materials. Results reported in [10] showed that in open-circuit condition, PVDF harvesters can produce higher peak-to-peak voltage than PZT harvesters, even if PZT harvesters are less sensitive to operating conditions (e.g., drop size, falling height). The impact of the raindrops on the harvester generates small and uneven amounts of electrical energy, which needs to be conditioned by a suitable power converter. A comparison between several circuits in terms of conversion efficiency and voltage ripple is presented in [7,11,12].

A raindrop harvester is a dynamic system excited by a series of impacts. Experimental results obtained with actual rain [13] and simulated rain [14] show that the rain drops generate series of well-separated force impulses on the harvester. Therefore, the study of the response of a harvester to a single raindrop is meaningful. The first studies on the impact of a raindrop were presented in [1]. Nowadays it is well recognized that there are several impact mechanisms (rebound, partial rebound, spreading, splashing). The boundaries between these different behaviors can be found by means of empirical equations based on experimental data [15], which are expressed in terms of dimensionless numbers (Weber and Reynolds numbers). An important feature of a piezoelectric harvester is that the first raindrops impact a dry surface. The following raindrops impact a wet surface and increase the water layer accumulated on the harvester surface. The water layer increases until it reaches the edges of the harvester and there is a spill. This unsteady behavior has some important effects on harvester dynamics [16,17].

The impact of a raindrop on the on the dry harvester surface leads to abrupt spreading and splashing with a waste of usable energy. Conversely, the presence of a water layer changes the impact dynamics, since the water layer accumulated on the harvester makes the impact surface softer [16]. The mass of water that accumulates on the surface of the harvester adds to the mass of the harvester itself and lowers the natural frequency of the system [14]. The water that accumulates on the harvester surface and that vibrates with the harvester increases the damping of the dynamic system [16].

When there is a water layer of sufficient depth on the harvester surface, the impact of raindrops generates water ripples that causes additional gravity loads on the harvester surface [17].

In a conventional cantilever harvester all these phenomena occur in a chaotic way, since the water layer on the harvester surface continuously changes and there are frequent and uncontrolled spills of water.

Since the previous studies [16] have shown that the presence of a water layer on the surface of a simple cantilever harvester has positive effects on harvester performance, the research presented in this paper aims to improve energy collection by creating a permanent water layer on the area impacted by raindrops. A novel raindrop harvester was developed, it is composed of a cantilever harvester and a rectangular-base spoon fixed on the cantilever tip. The impact area and the active piezoelectric layer are separated. The rectangular spoon, which is surrounded by small walls, creates a small pool so the raindrops always impact a liquid surface. The harvester with its electrical connections is protected from the raindrops by an enclosure. This design makes the functioning of the harvester more regular and efficient, since the raindrops always impact a small pool with a minimum depth, which is guaranteed by the walls of the spoon. The impact on the liquid surface generates a series of phenomena (liquid crater, liquid jet, and water ripples [18]) that contribute to harvester excitation.

In the next section of the paper, the advantages and drawbacks of the novel harvester are analyzed and discussed with the aid of numerical simulations. Then experimental equipment and methods are presented. Finally, experimental results in terms of open-circuit and on-load circuit voltage are presented and discussed, assessing the validity of the proposed design.

## 2. Theoretical Background

### 2.1. Impact Mechanics

**Figure 1:**
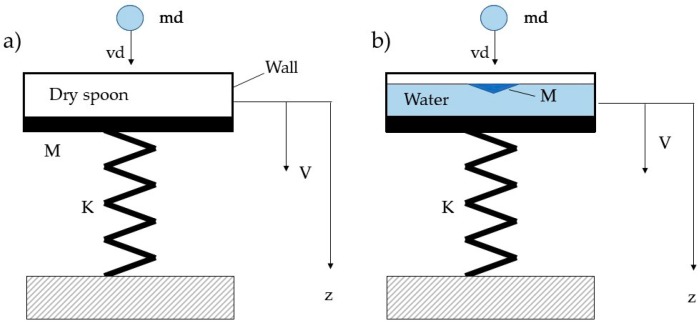
Raindrop impact on a dry (**a**) and wet (**b**) spoon; *v_d_* is raindrop velocity, *m_d_* raindrop mass, *V* spoon velocity.

Figure 1 shows the basic model for studying the impact of a raindrop with mass *m_d_* and velocity *v_d_* on a harvester. If the impact on a harvester equipped with a dry spoon is considered, mass *M* includes the mass of the spoon and the equivalent cantilever mass, which is a fraction of the total mass of the harvester (e.g., 23.6% of the total mass, if the first bending mode of a cantilever harvester is considered [19]). Parameter *k* is the stiffness of the cantilever beam. Since an impulse force with duration much smaller than the natural period of the vibrating system changes its initial velocity, but does not change its initial position [20], stiffness *k* is not relevant during the impact.

When the impact takes place on the solid surface of the spoon, the basic equations of impact mechanics [21] make it possible to calculate the final velocities of the spoon and of the drop (*V* and *v_d_*, respectively). They are the conservation of momentum (in the *z* direction) and the definition of coefficient of restitution (ε):

(1) $$m_{d}v_{d}=MV+m_{d}v_{d}^{\prime }$$

(2) $$v_{d}^{\prime }-V=-\varepsilon v_{d}$$

Then ratio $E^{*}$ between the kinetic energy transferred from the drop to the vibrating system and the kinetic energy of the impacting drop can be calculated:

(3) $$E^{*}=\frac{\frac{1}{2}MV^{2}}{\frac{1}{2}m_{d}v_{d}^{2}}$$

where the numerator is the maximum energy that the harvester could convert into electrical energy.

**Figure 2:**
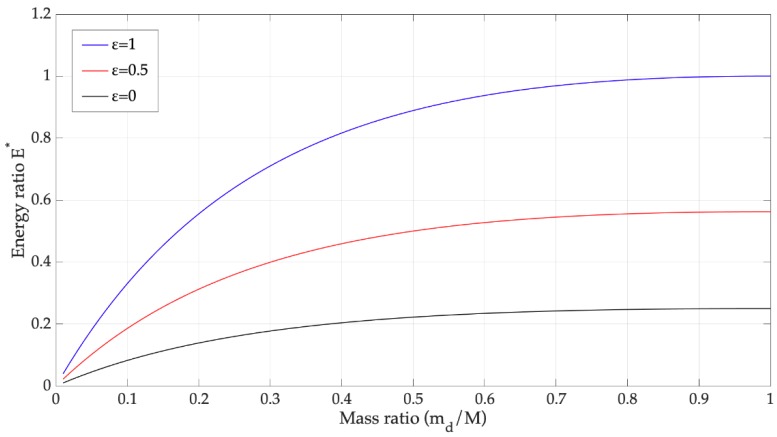
Ratio $E^{*}$ against ratio $\mu =m_{d}/M$ for some values of coefficient of restitution (ε).

Ratio $E^{*}$ is plotted in Figure 2 against ratio $\mu =m_{d}/M$ for some values of the coefficient of restitution (ε).

Since usually *m_d_* is a small fraction of *M*, a small amount of the input kinetic energy is transferred to the vibrating system and this amount decreases as *M* increases. An increase of ε  from 0 (inelastic impact) to 1 (elastic impact) increases the energy transfer to the vibrating system.

The condition that best approximates the impact of a raindrop on a dry harvester is an inelastic collision (ε=0) [1,22]. Therefore, with μ<0.01, the ratio between the transferred energy and the input kinetic energy is $E^{*}<0.01$.

The assumption of inelastic impact when the harvester surface is dry is corroborated by some experimental and calculated results. The impact of a drop on a dry solid surface is characterized by spreading, splashing, and rebound phenomena [22,23,24]. After the first phase of the impact, in which a shock wave is generated inside the drop [23], the liquid begins spreading out of the drop surface, generating a liquid lamella on the solid surface. The initial energy, which in the most general case includes kinetic energy $\left(E_{k}\right)$ and surface energy of the drop $(E_{s}$) during spreading is partially dissipated $\left(E_{d}^{\prime }\right)$, and partially transformed into surface energy $(E_{s}^{\prime }$); only a part of initial energy remains associated to the kinetic energy of the spreading lamella $\left(E_{k}^{\prime }\right)$, whose velocity is chiefly tangent to the solid surface. The following equation holds [23]:

(4) $$E_{k}+E_{s}=E_{k}^{\prime }+E_{s}^{\prime }+E_{d}^{\prime }$$

Recently, from numerical simulations, an energy balance during spreading was carried out [25] and results showed that roughly one half of initial kinetic energy is dissipated.

Splashing takes place when the drops disintegrate into secondary droplets after the impact on the dry solid surface. Splashing is characterized by the formation of a crown of liquid around the residual top of the impacting drop [24]. This crown may derive from the unstable rim of the liquid lamella during the last phase of spreading [23]. After crown formation, droplets are ejected from the rim of the crown. The splashing mechanism is not very efficient from the point of view of energy harvesting, since the spreading phase of the lamella is characterized by large energy dissipations, and the kinetic energy of the droplets that spread in every direction can only be partially exploited [16].

A rebound or partial rebound of the drop can take place at the end of the spreading phase. After reaching the maximum extension, the lamella of liquid begins receding. If dissipation is not too large, the residual energy is converted into kinetic energy again, when the shrinking lamella approaches the contact point, residual energy may be sufficient to eject some liquid upwards from the surface [24,26].

Given the physical properties of water (density ρ = 1000 kg/m^3^, surface tension σ = 0.07 N/m^2^, and dynamics viscosity μ_d_ = 8.9 × 10^−4^ Ns/m^2^), raindrop diameter (*d_d_*), and speed (*v_d_*), it is possible to analyze the impact conditions of a raindrop on a dry solid surface by means of empirical equations that fit experimental data [1,15,16]. The impacting raindrop tends to splash if this condition is satisfied [1]:

*We*^0.5^*Re*^0.25^ > 57.7, (5)

in which *We* is the Weber number (*We =* *ρd_d_v_d_^2^/σ*) and *Re* the Reynolds number (*Re =* *ρd_d_ v_d_/*μ_d_). Actually, in most of the reported cases [16,22,27], the impact of raindrops on a harvester leads to splashing phenomena with large energy dissipations that correspond to inelastic impacts. These results are in agreement with the small conversion efficiencies reported in [5,27].

The scenario drastically changes when the impact of a raindrop on a liquid surface is considered. In this case only a part of the water inside the spoon is directly impacted by the raindrop, its mass (*M* in Figure 1b) can be smaller than the sum of spoon mass plus the equivalent cantilever mass. Therefore, the basic impact model (Figure 2) suggests that the impact on a liquid surface causes an increase in the ratio μ with a positive effect on collected energy.

The impact of a drop on a liquid surface is rather different from the impact on a solid surface [23,28,29,30]. The impacting drop creates a crater on the liquid surface whose geometry can be approximated as a spherical cavity [28]. The maximum extension of the crater can be estimated with an energy approach [29,30] in which the impact energy $\left(E_{k}+E_{s}\right)$ is converted into gravity potential energy ($E_{p}^{\prime }$) related to the raising of the liquid surrounding the crater, and into surface energy of the crater ($E_{s}^{\prime }$). It is worth noticing that references [29] and [30] neglect energy dissipation during the formation of the crater and this equation holds:

(6) $$E_{k}+E_{s}=E_{s}^{\prime }+E_{p}^{\prime }$$

After reaching the maximum extension, the crater begins collapsing, capillary waves distort the shape of the crater which assumes the shape of an “amphitheater” [30]. In this phase the liquid accelerates upwards and energy is converted into kinetic energy again. When the crater collapses, a central liquid jet is ejected from the liquid surface. Finally, the liquid jet pinches-off and generates secondary drops that impact again the liquid surface.

The duration of this sequence of phenomena is some tens of ms, and it is much longer than the duration of the dry impact of a drop (with diameter *d_d_*), which is usually estimated with the formula [16]:

*τ* = *d_d_*/*v_d_* (7)

In the impact with a liquid surface, gravity plays an important role and in the final phase of the phenomenon (jet formation) the surface and gravity energy are converted into kinetic energy again. For these reasons, the impact on the liquid surface potentially transfers more energy to the system that can be converted into electric energy by the harvester.

### 2.2. Raindrop Harvester Vibrations

Usually cantilever harvesters excited by raindrops are simulated, taking into account only the first mode of vibration of the system [31,32]. Multi-mode dynamic models of cantilever harvesters have already been presented in the scientific literature, but they refer to the case of a harvester excited by base vibrations [33,34,35]. In that case, the cantilever is loaded by the distributed inertia force caused by the distributed mass of piezoelectric and structural materials and by the lumped inertia force caused by the tip mass. The presence of the tip mass increases the excitation and the generated voltage in open-circuit and on-load circuit conditions [36,37].

A raindrop harvester with spoon has a rather different dynamic behavior. The water is a tip mass, but the system is excited by impulse forces applied to the spoon, therefore the added mass due to water does not increase the excitation of the system, conversely it reduces the mobility of the free-end of the harvester and increases damping.

The raindrop harvester with a spoon was developed starting from a Midé PPA 1001 unimorph cantilever harvester. The cantilever is made of a single piezoelectric layer for converting mechanical vibrations into electrical energy and of a steel laminate for system stiffness—details about harvester properties are given in Appendix A. The piezo-MEMS is clamped at one end to a fixed base and is integral to a spoon filled by water at the free-end.

**Figure 3:**
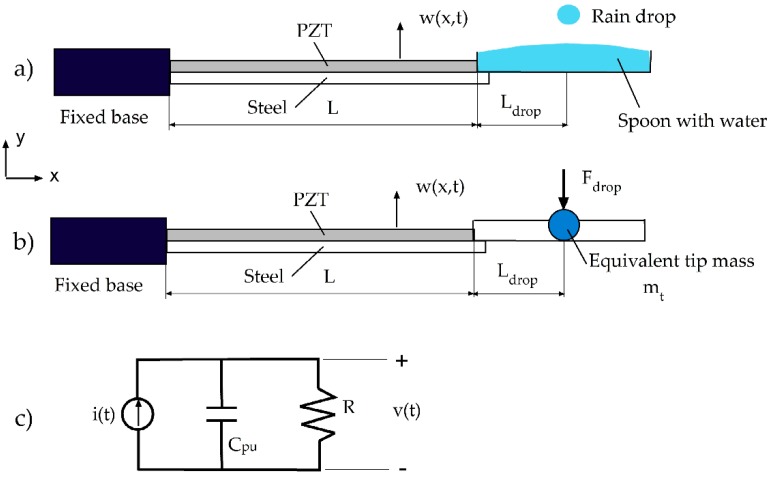
Mathematical model of the harvester with spoon; (**a**) full mechanical model with spoon; (**b**) equivalent mechanical model; (**c**) equivalent electric circuit.

The dynamic behavior of the harvester with a spoon hit by a raindrop is schematized according to Figure 3.

Since the aim of the study is the evaluation of the effect of the added mass on the dynamic response of the harvester, an ideal inelastic impact [16] is considered. The impact force is represented by this equation:

(8) $$F_{drop}\left(t\right)=\frac{m_{d}v_{d}}{\tau }\;\left(H\left(t\right)-H\left(t-\tau \right)\right)$$

where H(t) is the Heaviside step function, *m_d_* = 0.0042 g, *v_d_* = 4 m/s, and *τ* = 0.5 ms.

The effect of the spoon, filled with water, is simulated by a tip mass $m_{t}$ with inertia moment $I_{t}$. The concentrated force can be then transported to the cantilever end (x=L) by a pair of opposite forces $F\left(t\right)=F_{drop}\left(t\right)\;L_{drop}/h$, placed at fictitious distance h. In such a way, a distributed parameter model of the unimorph cantilever harvester with a spoon can be derived, such as the tip mass model developed by Inman and Erturk [19,34].

The forced vibration equation for the transverse displacement of the cantilever w(x,t) becomes:

(9) $$EI\;\frac{\partial^{4}w\left(x,t\right)}{\partial x^{4}}+c_{s}I\;\frac{\partial^{5}w\left(x,t\right)}{\partial x^{4}\partial t}+m\frac{\partial^{2}w_{r}\left(x,t\right)}{\partial t^{2}}+c_{a}\;\frac{\partial w\left(x,t\right)}{\partial t}+\theta v\left(t\right)\;=\left(F_{drop}\left(t\right)+F\left(t\right)\right)\;\delta \left(x-L\right)-F\left(t\right)\;\delta \left(x-\left(L-h\right)\right)$$

where EI is the bending stiffness, m the mass per unit length of the cantilever, and I the equivalent area moment of inertia of the composite section. The strain rate damping coefficient $c_{s}$ and the air damping coefficient $c_{a}$ are assumed to satisfy the proportional damping criterion in order to obtain a much easier application of modal expansion approach for the solution of the partial derivative equation (PDE). The coupling with the electric problem, depending on voltage v(t), is by the backward piezoelectric coefficient θ. Terms at the right-hand side of Equation (9) (where δ(x) indicates the Dirac delta) are concentrated force impulses applied to x=L and x=L−h, respectively.

The unimorph cantilever is typically connected to an external electrical load (e.g., a sensor fed by a power conditioning unit), which can be schematized by a resistance R. The charge generation from the piezoelectric effect is represented as an equivalent current source i(t), whereas the capacitive effect related to the electric field in the piezo-layer is simulated by an equivalent capacitance $C_{pu}$ (see Figure 3c). The electrical dynamics of a piezo-MEMS can be thus described by a first-order R-C circuit, which is governed by the following ordinary differential equation (ODE):

(10) $$C_{pu}\frac{dv\left(t\right)}{dt}+\frac{v\left(t\right)}{R}=i\left(t\right)$$

According to the modal expansion method, natural frequencies and eigenfunctions (i.e., vibration modes) are first obtained by resolving the equation of free-vibrations:

(11) $$EI\;\frac{\partial^{4}w\left(x,t\right)}{\partial x^{4}}+m\frac{\partial^{2}w\left(x,t\right)}{\partial t^{2}}=0$$

after imposing clamped end at x=0 and inertia force and torque corresponding to the tip mass at x=L.

The displacement along the cantilever can be thus expanded by an absolutely and uniformly convergent series of vibration modes $\phi_{i}$, as

(12) $$w\left(x,t\right)=\overset{\infty }{\underset{i=1}{\sum }}\;\phi_{i}\left(x\right)\;\eta_{i}\left(t\right)$$

By inserting Equation (12) in Equation (11), vibration modes $\phi_{i}$ are obtained. These can be used also for expanding the displacement of Equation (9), due to the proportional damping assumption.

The i-th modal response $\eta_{i}$ can be found solving the second-order ODE:

(13) $$\frac{d^{2}\eta_{i}\left(t\right)}{dt^{2}}+2\zeta_{i}\omega_{i}\frac{d\eta_{i}\left(t\right)}{dt}+\omega_{i}^{2}\eta_{i}\left(t\right)+\chi_{i}v\left(t\right)=f_{i}\left(t\right)$$

with modal force $f_{i}\left(t\right)=\;F_{drop}\left(t\right)\;\phi_{i}\left(L\right)+F\left(t\right)\left(\phi_{i}\left(L\right)-\phi_{i}\left(L-h\right)\right)$. $\omega_{i}$ is the undamped natural angular frequency of the i-th mode and $\zeta_{i}$ is the damping ratio of the i-th mode. The backward coupling coefficient in Equation (13) is defined as in [19]:

(14) $$\chi_{i}=\theta \frac{d\phi_{i}\left(L\right)}{dx}$$

After solving the modal equation system, the source electric current can be expanded by using modal responses as

(15) $$i\left(t\right)=\overset{\infty }{\underset{i=1}{\sum }}\varphi_{i}\frac{d\eta_{i}\left(t\right)}{dt}$$

where $\varphi_{i}=-e_{31}h_{pz}b$ $\frac{d\phi_{i}\left(L\right)}{dx}\;$ is the forward modal coupling term, with $e_{31}$ piezoelectric constant, $h_{pz}\;$ thickness of piezoceramic layer, and b is the width of the beam.

If the first N modes are considered, the final system is made by of N second-order ODEs (i.e., modal equations) coupled with a first-order ODE, i.e., an external circuit equation. This system is recast in a state-space form, which is suitable for numerical solution under Matlab^®^ software environment:

(16) $$\{\begin{array}{c} \dot{x}=Ax+Bu\\ y=Cx+Du \end{array}$$

where system matrices are defined as

(17) $$A=\left[\begin{array}{ccc} O_{N,N} & 1_{N,N} & O_{N,1}\\ -\Omega^{2} & -2\zeta \Omega & \chi \\ O_{1,N} & \varphi^{T}/C_{pu} & 1/\tau \end{array}\right],B=\left[\begin{array}{c} O_{N,1}\\ 1_{N,1}\\ O \end{array}\right],C=\left[\begin{array}{ccc} 1_{N,N} & O_{N,N} & O_{N,1}\\ O_{N,N} & O_{N,N} & O_{N,1}\\ O_{1,N} & O_{1,N} & 1 \end{array}\right],D=\left[\begin{array}{c} O_{N,1}\\ O_{N,1}\\ O \end{array}\right],$$

and system vectors are

(18) $$x={\left[\eta_{i}\;\dot{\eta_{i}}\;v\right]}^{T},\;y={\left[\eta_{i}\;O_{1,N}\;v\right]}^{T},\;u={\left[O_{1,N}\;f_{i}\;0\right]}^{T}$$

with index i=1,…,N. $O_{N,N}$ is the null matrix, $1_{N,N}$ is the identity matrix, $\Omega =\left[\omega_{i}\right]$, $\zeta =\left[\zeta_{i}\right]$ are diagonal matrices, and $\varphi =\left[\varphi_{i}\right]$, $\chi =\left[\chi_{i}\right]$ are column vectors, all of size *N*. $O_{N,1}$ and $1_{N,1}$ are column vector of size *N*, with null and one coefficients, respectively. Superscript *T* indicates the matrix transpose. $\tau =RC_{pu}$ is the time constant of the equivalent electrical circuit.

First, the mathematical model of Figure 3 was used to calculate the modal properties (natural frequencies and modes of vibration) of the harvester with an empty spoon (added mass 1.07 g) and with a filled spoon (added mass 4.5 g), and the results were compared with those of the harvester alone.

**Figure 4:**
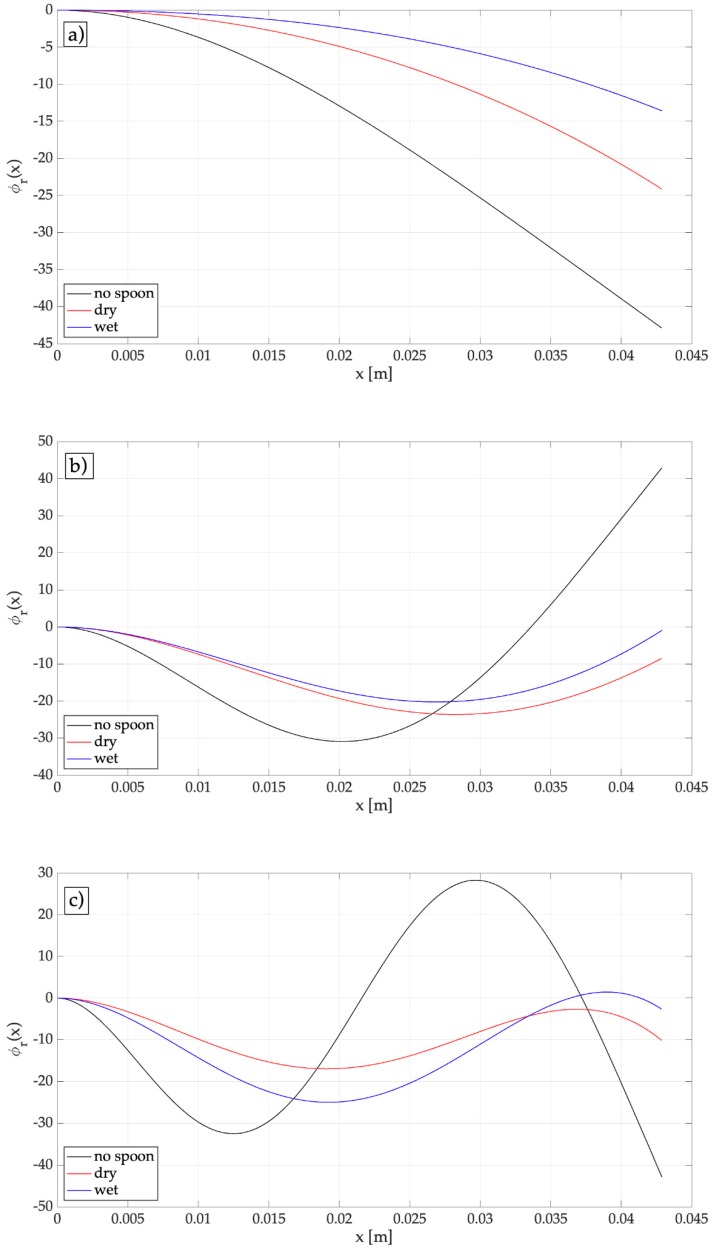
Mass-normalized modes of vibration: (**a**) First-order, (**b**) second-order, (**c**) third-order; dry: empty spoon, wet: filled with water.

The added mass has a large effect on the natural frequencies, since the fundamental frequency lowers from 118 to 54 Hz, owing to the added mass of the empty spoon, and lowers to 31 Hz owing to the added mass of the filled spoon. Figure 4 shows that the added mass has a relevant effect on the modal shapes. The second and third modes of vibration do not show a vibration anti-node at the free-end of the harvester.

**Figure 5:**
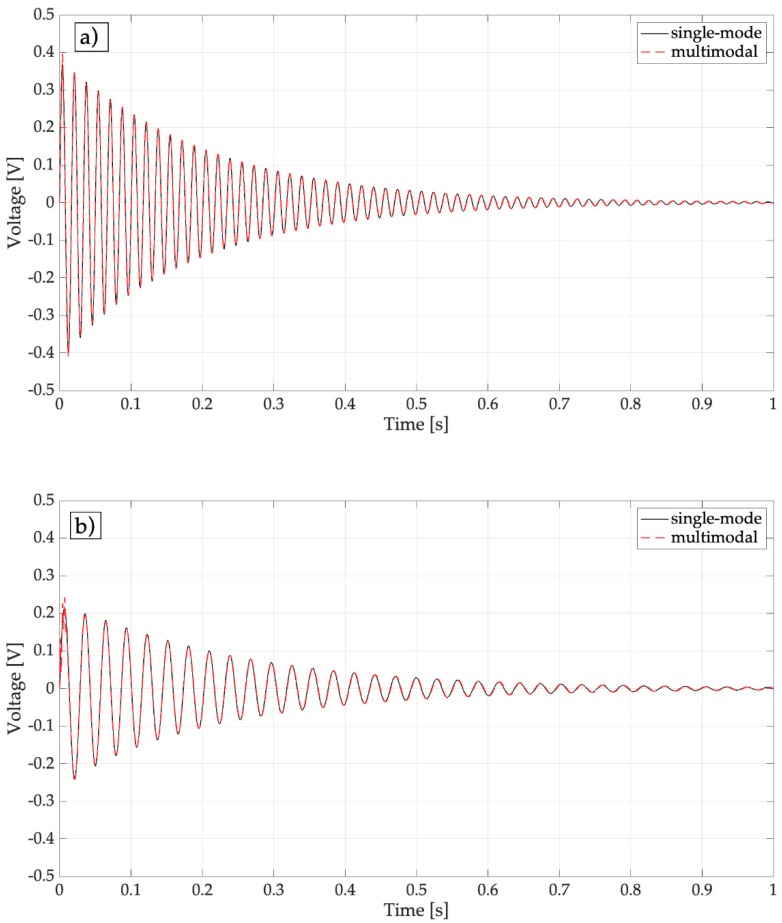
Effect of added mass on the peak-to-peak generated voltage: (**a**) Harvester with an empty spoon; (**b**) effect of the added water mass; single-mode: only the first-mode is considered, multimodal: the overall effect of the first three modes is considered.

Then the forced response of the system stimulated by the finite width rectangular impulses of Equation (8) was simulated and the open-circuit voltage was calculated. Figure 5 shows the voltages calculated by means of the multi-mode model (3 modes) and by means of the single-mode model.

**Figure 6:**
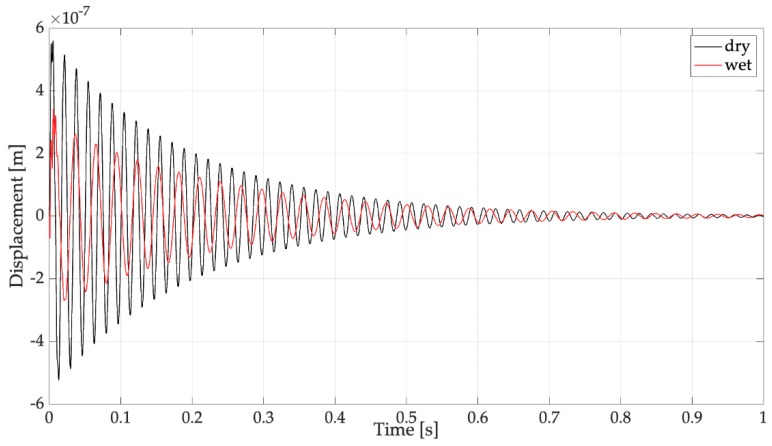
Effect of added mass on the cantilever tip displacement (dry: harvester with empty spoon, wet: spoon filled with water).

There are very small differences between the results of multi-mode and single-mode simulations that take place in the first instants of the response. The time histories are damped oscillations (like in the presence of a Dirac impulse), because the duration of the impulse (*τ* = 0.5 ms), which was calculated according to Equation (7), is much smaller than the natural period of the first mode of the harvester (18.5 ms with an empty spoon and 32.2 ms with a filled spoon, respectively). Figure 5 highlights that the added mass due to water inside the spoon has a negative effect on the dynamics of the harvester, because the generated voltage roughly halves with respect to the condition of empty spoon. Figure 6 shows that this phenomenon takes place because the added mass reduces the mobility of the free-end of the harvester and thus the strain level inside the piezoelectric layer.

In conclusion, it is possible to state that the analysis of impact mechanics shows a positive effect on the performance of the harvester due to the impact on a liquid surface. Conversely, the study of harvester vibrations highlights a negative effect of the added water mass. In order to understand what the dominant effect is, a prototype was built and experimental tests were carried out.

## 3. Development and Testing of the Prototype

**Figure 7:**
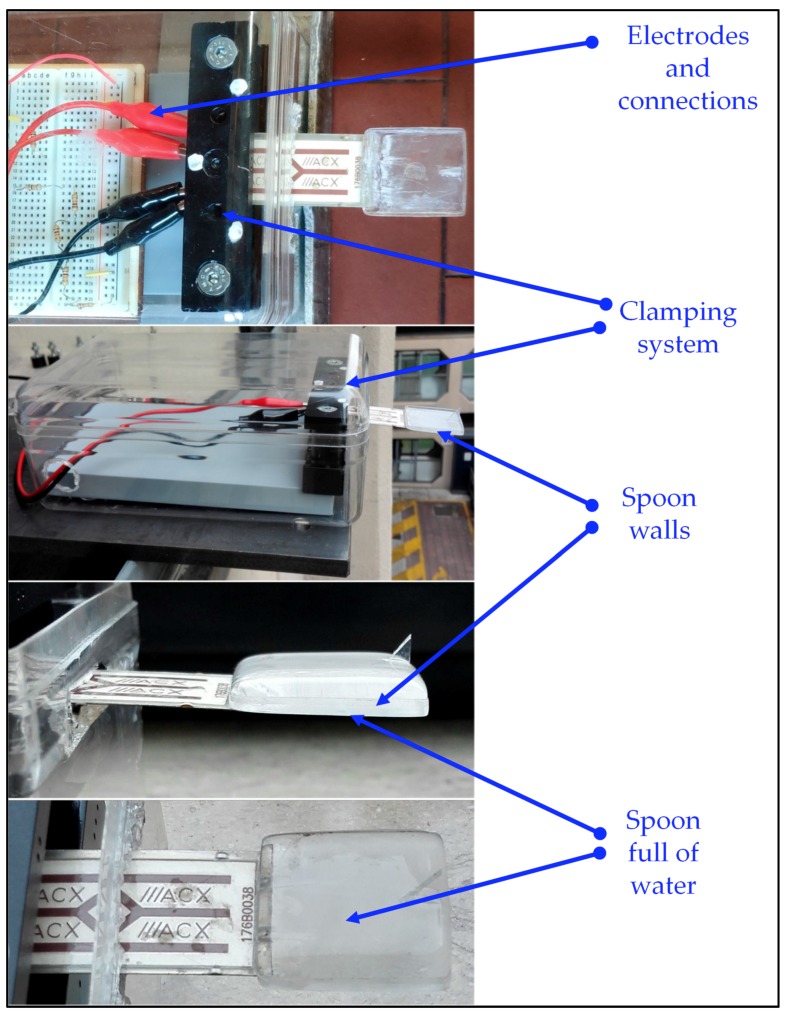
Prototype of the raindrop harvester with spoon.

Nowadays, commercial cantilever piezoelectric harvesters are cheap, sturdy, and versatile devices. Therefore, the prototype of a harvester with a spoon was developed, starting from a Midé PPA 1001 unimorph cantilever harvester having a rated natural frequency of 125 Hz. This harvester was chosen because its properties were well known, since it had been extensively tested in the framework of previous researches [36,38]. The natural frequency of this device depends on the position and stiffness of its clamping. For this reason, a proper clamping system was developed. It was composed of two small steel blocks that were pressed against the clamping area of the cantilever by means of external bolts. The spoon had square shape (29 × 29 mm) and small edges (1 mm), it was connected to the free-end of the harvester by means of cyano-acrylate glue, the mass of the empty spoon was 1.07 g. The overall dimensions of the harvester with spoon were: Length 82.0 mm, width 29.0 mm, and height 2.5 mm. The cantilever harvester with its clamping system was enclosed into a plastic box, to protect the electric system from raindrops and humidity. Figure 7 shows the prototype of the raindrop harvester. The low hydrophilicity of water on a plastic surface produces an increase in the water contained in the spoon, thus the mass of the spoon full of water was 4.50 g.

**Figure 8:**
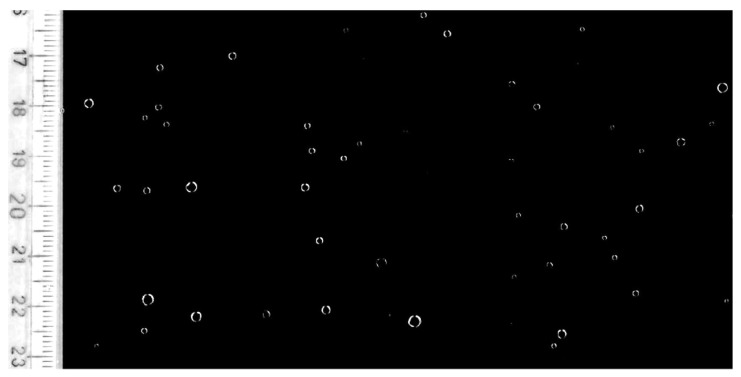
Raindrops collected on a plastic sheet.

**Figure 9:**
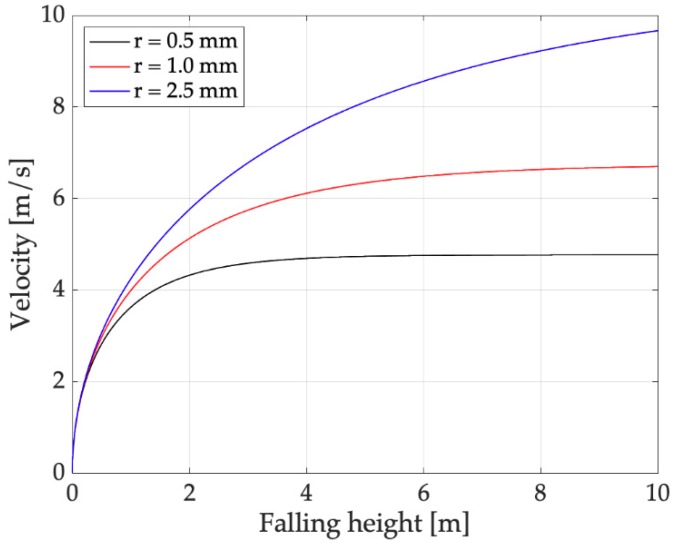
Raindrop velocity against falling height (the asymptotic value is the limit velocity).

For performing laboratory tests, a reference raindrop has to be defined. Actual raindrops were collected and photographed on black plastic sheets during a mild rain in Padova, see Figure 8. The processing of the photographs made it possible to determine a maximum drop radius ($r_{d}$) of about 1 mm, which corresponds to a mass $m_{d}=0.0042\;g$. The motion of these raindrops can be studied solving a simple ODE [5,16] and the limit speed can be calculated. Results, which are represented in Figure 9, showed that with $r_{d}=1\;\mathrm{mm}$, the limit speed is 6.7 m/s. With a falling height of 1 m, which can be easily obtained in a laboratory test, the final speed is $v_{d}=4\;m/s$, which is a substantial fraction of the limit speed. These are the parameters of the reference raindrop.

**Figure 10:**
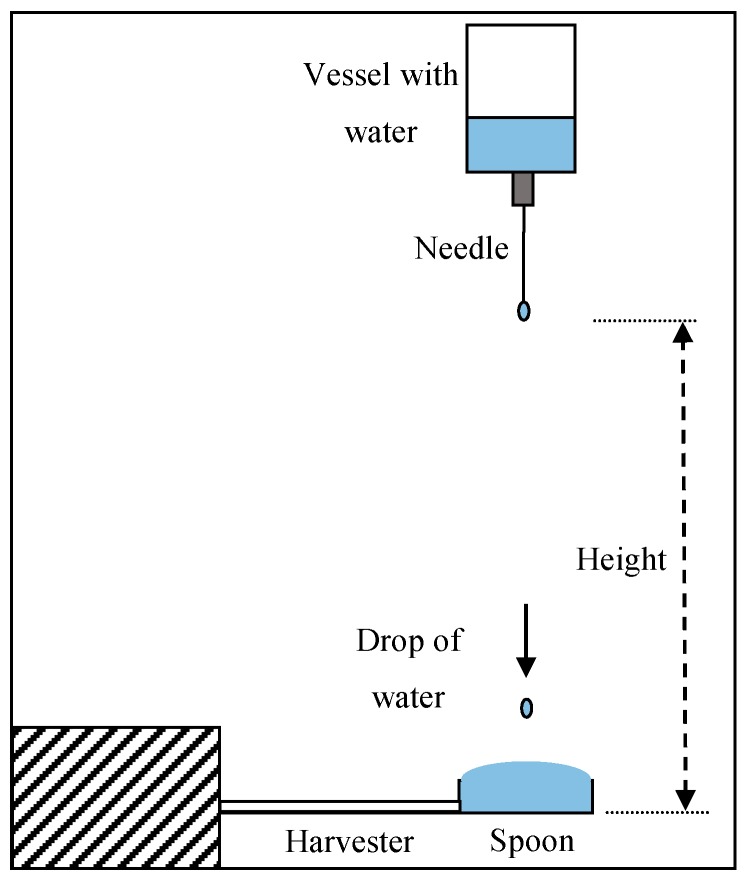
Experimental equipment.

Drops of water were produced by a vessel located 1 m above the harvester, having a hole on the bottom connected to a needle, see Figure 10. A standard needle that produces drops with an average radius of 1 mm was used. The system was carefully set up so that the raindrop hit the center of the spoon.

The voltage signal generated by the harvester was acquired by means of a LabVIEW^®^ system composed of a NI9243 acquisition module and a proper software able to show both the time and frequency domain responses. A trigger was set in order to acquire the response due to the impact of a single water-drop falling on the spoon. Typically, four thousand samples were acquired at a sampling frequency of 2 kHz. This sampling frequency was suited to the measurements, since numerical simulations and some preliminary tests showed that harvester vibrations caused by impacts are dominated by the first modes of vibrations that have natural frequencies much lower than 1000 Hz.

The following series of tests was performed; each series consisted of at least five trials to verify the repeatability of the system.

- Filled spoon or dry spoon.
- Dry spoon with rigid masses corresponding to water mass.
- Open-circuit and on-load circuit with resistive load.

The effect of raindrop impacts on a simple cantilever harvester was not analyzed, because the absence of the spoon reduces the moment of the impact force on the cantilever and a comparison with the harvester with a spoon is not possible.

## 4. Experimental Results

### 4.1. Open-Circuit Voltage

**Figure 11:**
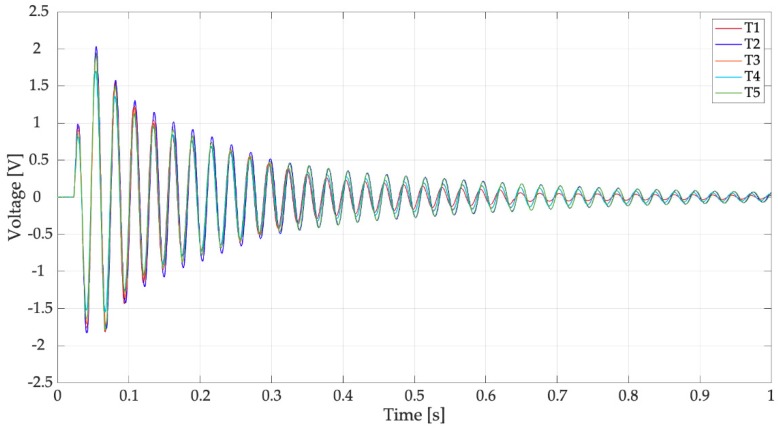
Tests with drops falling from 1 m on the filled spoon, open-circuit voltage.

The first tests were carried out without any electrical load. The only resistance in the electric circuit (*R* in Figure 3c) was the resistance of the data logger, which was very large (305 kΩ). Figure 11 shows the voltage generated by the harvester equipped with the spoon full of water when hit by reference drops falling from 1 m. In this test condition, the effects of the impact on a liquid surface (e.g., water ripples) were taken into account. In Figure 11, five different trials are considered, which are named T1, T2, T3, T4, and T5; the results are very repeatable. The waveform of generated voltage is roughly a damped oscillation of a system dominated by one mode of vibration. Excluding the first oscillations, the effect of higher order modes is negligible. Experimental data were elaborated numerically to calculate mean values of oscillation frequency, damping ratio, and peak-to-peak voltage.

The mean frequency of the trials was 37.01 Hz with a standard deviation of 0.06 Hz. This frequency value was not far from the value estimated by the numerical model that considers water as a lumped mass (31 Hz). The damping ratio of the system (ζ) was calculated with the logarithmic decrement method [20], which is based on the assumption of a linear viscous damping. This assumption usually is well verified by a standard harvester in open-circuit condition, but may be less sound for a raindrop harvester due to the motion of water inside the spoon [14]. Actually, the values of ζ calculated using the first part of the signal (t < 0.3 s), which are characterized by the largest amplitudes, are significantly larger than the values obtained, considering the rest of the signal. This is a non-linear effect due to the presence of water. The mean damping ratio of the trials was 0.015 with a standard deviation of 0.002.

An important feature of the responses of Figure 11 is that the first peak is not the highest, as in the response of a second order system excited by a finite-width impulse with duration *τ* much smaller than the natural period. This effect is caused by the presence of water in the spoon that modifies impact dynamics. The duration of the voltage waveform appears much longer than first phase of the response that is influenced by the impact and motion of water inside the spoon. The mean value of peak-to-peak open circuit voltage is 3.22 V with a standard deviation of 0.68 V.

**Figure 12:**
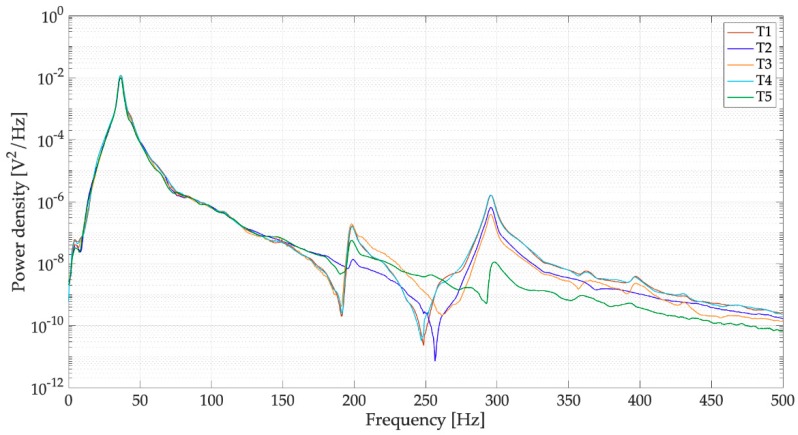
Tests with drops falling from 1 m on the filled spoon, power spectral densities (PSDs) of open-circuit voltage.

The power spectral densities (PSDs) of the voltages of Figure 11 are represented in Figure 12. In the frequency domain, the response is largely dominated by peak of the first mode (37.01 Hz), the contribution of the higher order modes of vibration (200 and 296 Hz) is very small. The different trials sometimes show different values of the peaks of the higher order modes. This phenomenon happens because there are variations in the drop impact position and this parameter influences the excitation of the higher order modes more than the excitation of the first mode. It is worth noticing the presence of a small peak at low frequency (3.5 Hz), which can be associated to water sloshing.

**Figure 13:**
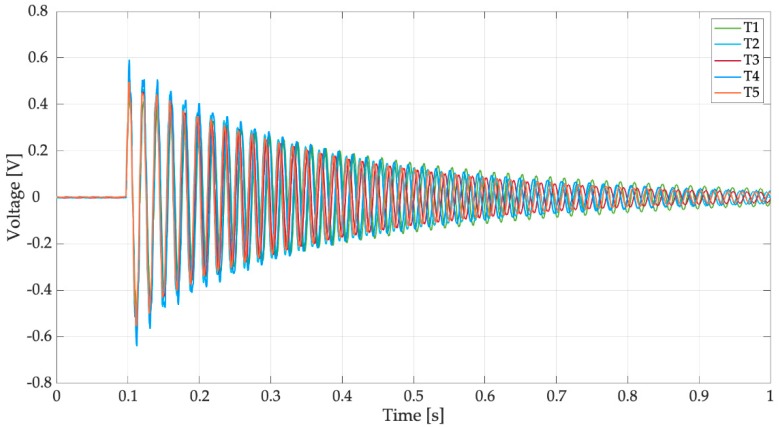
Tests with drops falling from 1 m on the empty spoon, open-circuit voltage.

Figure 13 shows the voltage generated by the harvester when the raindrops fall on the dry spoon, five different trials are considered (T1, T2, T3, T4, and T5) and after each trial the spoon was dried. The mean frequency of oscillation is 52.09 Hz with a standard deviation of 0.66 Hz. This value is very close to the numerical one (54 Hz). Damping ratio ζ, calculated with the logarithmic decrement method, had a significantly lower result than in the previous case: ζ = 0.008, with a standard deviation of 0.001. It is worth noticing that in this case the behavior of the system is more linear, since there is a very small difference between the values of ζ, identified using the highest peaks of the response (t < 0.3 s), and the values obtained using the rest of the signal.

With a dry spoon, the first peak of the response is the highest and the peak-to-peak voltage has a mean value of 1.03 V with a standard deviation of 0.17 V. This value is much smaller than the one obtained with a full spoon and confirms the beneficial effect of the water layer. The comparison between Figure 13 and Figure 5a confirms the validity of the numerical model, when the inelastic impact on a dry surface is simulated.

**Figure 14:**
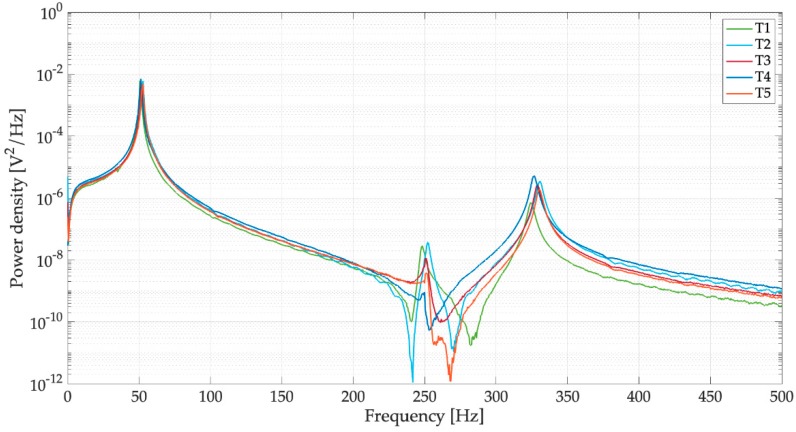
Tests with drops falling from 1 m on the empty spoon, PSDs of open-circuit voltage.

**Figure 15:**
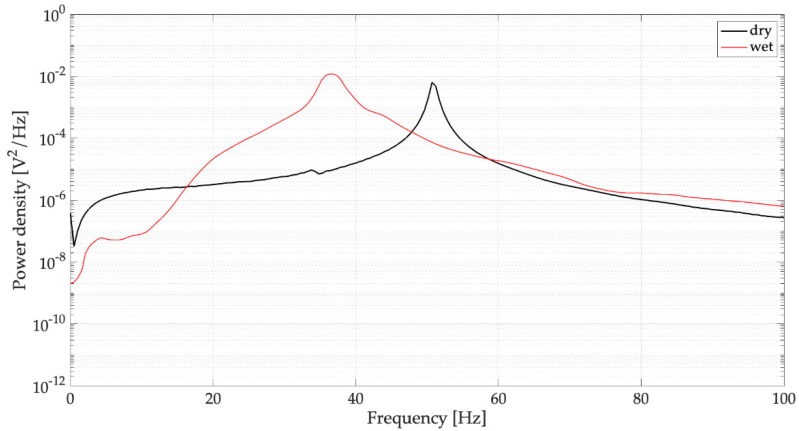
Effect of the filled spoon on the PSDs of open-circuit voltage (dry: harvester with empty spoon, wet: spoon filled with water).

The PSDs of the voltages generated by the dry harvester are represented in Figure 14 and corroborate the effects of water removal that were highlighted by the time domain analysis. The comparison in the low frequency band (0–100 Hz) between two typical PSDs obtained with full and dry spoons (Figure 15) shows that the main peak has a smaller bandwidth around the resonance; this result is in agreement with the decreased damping. The small peak at low frequency related to water sloshing disappears.

To confirm the large beneficial effect of the impact on the liquid surface and the negative effect of water mass, further specific experiments were carried out.

**Figure 16:**
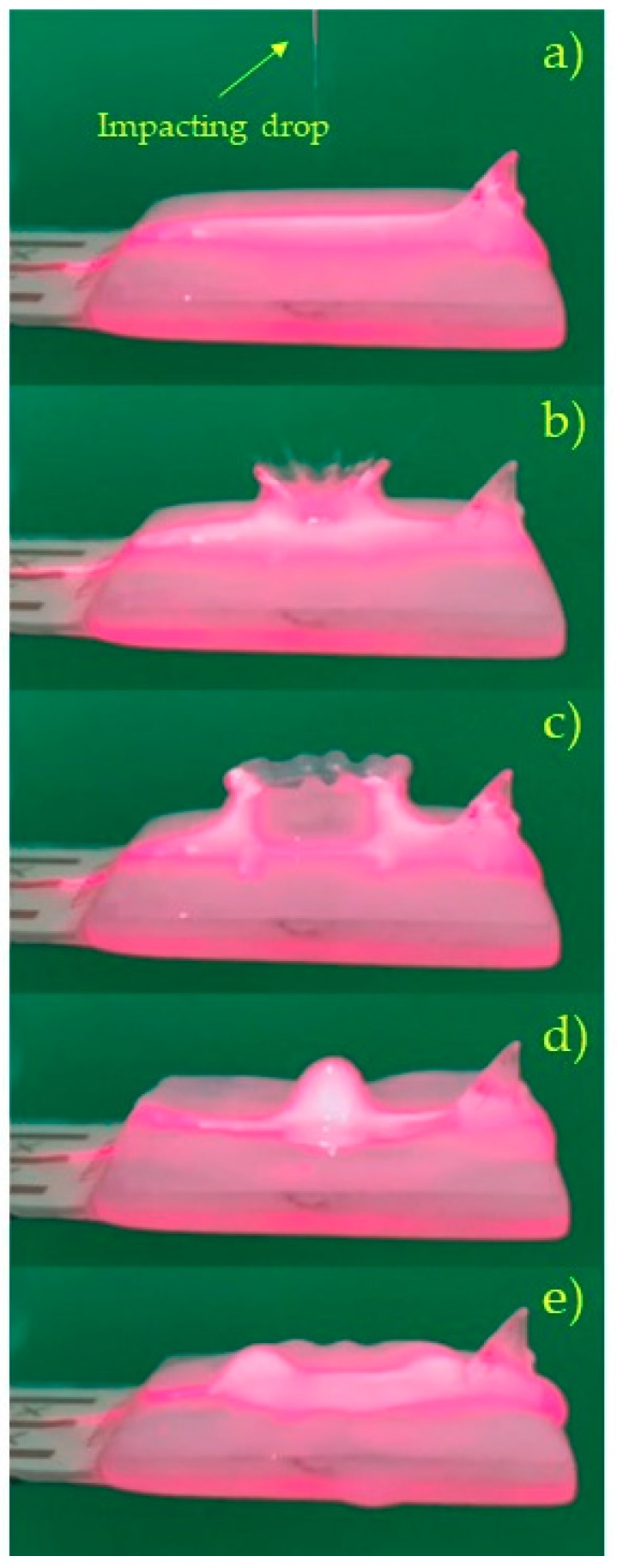
Sequence of four pictures of the spoon full of water taken just after the impact of a raindrop.

During some trials of the impact of raindrops on the liquid surface of a filled spoon, sequences of pictures were taken. A laser blade was used to highlight a section of the profile of the liquid surface. An example of these pictures is shown in Figure 16. The small fin that appears at the right side of the pictures is integral with the spoon and was used for exciting the system with a hammer in preliminary modal tests.

The pictures of Figure 16 in agreement with [30] show: The impacting drop (a), the formation of the crater (b), the “amphitheater” (c), the collapse of the crater with the formation of the secondary drop (d), the sloshing waves (e). These phenomena are able to improve the collection of energy.

Then, in a series of tests, water was removed from the spoon and solid masses were fixed under the dry spoon. The purpose of these tests was to analyze only the effect of the added mass without changing the impact phenomena. In all the tested cases, owing to the impulsive excitation, the harvester with added mass vibrated at its natural frequency [20]. Results in terms of mean peak-to-peak voltage are summarized in Table 1.

If the water mass (3 g) is replaced by an equivalent solid mass, there is a large decrease in the generated voltage from 3.22 V to 0.65 V. It is worth noticing that the voltage generated by the dry harvester with added solid mass was lower than the voltage generated by the dry harvester (1.03 V), this result is in agreement with numerical results presented in Section 2.2. If the added mass is further increased (6.3 g), the generated voltage further decreases.

Thus, the large positive effect of the impact on the liquid surface of the filled spoon exceeds the negative effect of the added mass.

### 4.2. On-Load Voltage

The open-circuit voltage of the harvester is an important figure [39] for the design of the rectifying circuit [7], nevertheless the on-load voltage $V_{c}\left(t\right)$ is more important, since it is related to the energy harvested by the device. If a simple resistive load is considered, the collected energy is

(19) $$W=\frac{1}{R_{L}}\int_{0}^{t_{max}}V_{c}{\left(t\right)}^{2}dt$$

in which $R_{L}$ is the load resistance and [0 $t_{max}$] is the interval of time considered for energy calculation. Since the generated voltage is a damped oscillation, a reference final time $t_{max}$ equal to thirty periods of oscillation was considered, because after this time the signal was almost extinguished. In an on-load condition, the overall resistance (R) represented in Figure 3c is the result of the parallel connection of data logger resistance ($R_{D}=\;$305 kΩ) and of load resistance $R_{L}$.

(20) $$R={\left(\frac{1}{R_{L}}+\frac{1}{R_{D}}\right)}^{-1}$$

Load resistance $R_{L}$ was chosen in order to make R equal to the optimal resistance [39] that maximizes the power generated by the harvester with capacitance $C_{pu}$ and natural frequency $\omega_{n}$, which in this case coincides with the natural frequency of the first mode of vibration that dominates the harvester response:

(21) $$R=\frac{1}{\omega_{n}C_{pu}}$$

**Figure 17:**
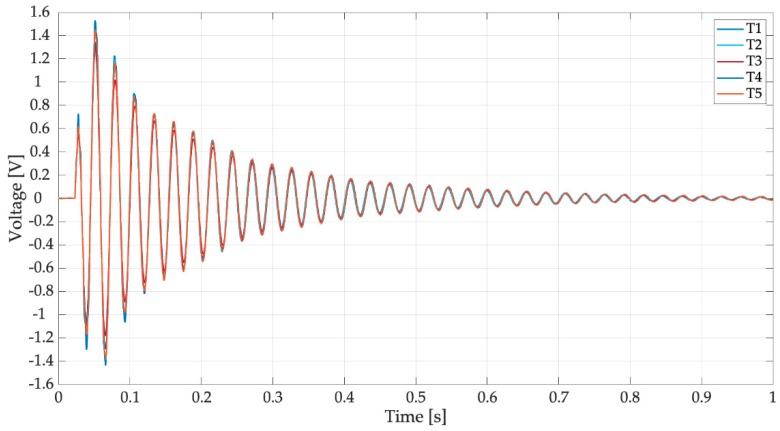
Tests with drops falling from 1 m on the filled spoon, on-load voltage.

**Figure 18:**
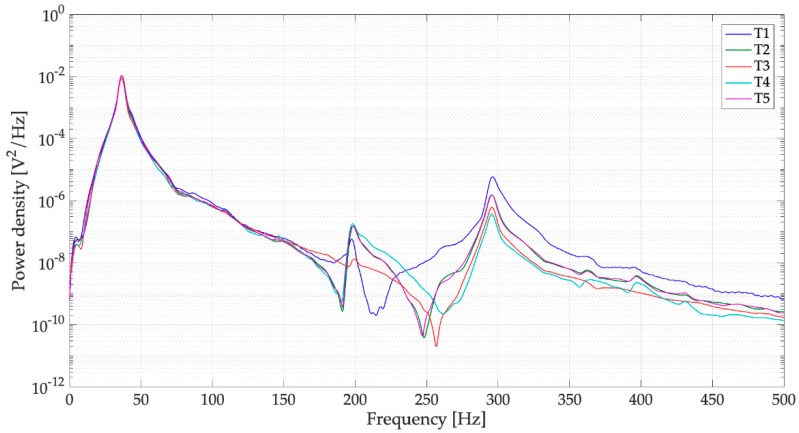
Tests with drops falling from 1 m on the filled spoon, PSDs of on-load voltage.

Figure 17 and Figure 18 shows the results of five trials (T1, T2, T3, T4, and T5) carried out with the full spoon and with the optimal load resistance (44.2 kΩ). The trials are very repeatable and the waveforms and PSDs are similar to the one measured in an open-circuit condition (Figure 11 and Figure 12).

The analysis of measured signals highlights some interesting features of the on-load response. The frequency of oscillation was 36.49 Hz (mean value) with a standard deviation of 0.1 Hz. This value is a bit lower than the one measured in open-circuit (37.01 Hz), as happens in the harvesters excited by base vibrations [19,39]. The damping ratio was 0.021 (mean value) with a standard deviation of 0.001, this value is larger than the one measured in open-circuit (0.015), since the load resistance contributes to damping [19]. Finally, the generated voltage decreases with respect to the open-circuit voltage, the mean peak-to-peak value was 2.48 V with a standard deviation of 0.24 V. This result agrees with the ones found for harvesters excited by base vibrations [33,38]. Starting from Equation (19), the collected energy was calculated and a mean value of 1.58 μJ was obtained (with a standard deviation of 0.23 μJ). This value is roughly one order of magnitude larger than the one obtained with a dry spoon and larger than most of the values reported in the literature [2,5,27], see Table 2. It is worth noticing that the comparison with literature values is not easy, owing to the different test conditions and electrical loads.

## 5. Conclusions

The analysis of the scientific literature dealing with drop impact dynamics has shown that, if a raindrop harvester is equipped with a liquid surface, the amount of energy that is collected from a raindrop impact can be increased. Conversely, numerical simulations have shown that the added water mass has a negative effect on the harvester performance, because it reduces the mobility of the cantilever. In order to investigate these phenomena, a prototype harvester with a spoon full of water for collecting raindrops on a liquid surface was developed.

The experimental tests carried out on the prototype highlighted that when the impact takes place on the liquid surface of the spoon, there is an improvement in the generated voltage. Therefore, the negative effect of the added water mass that is foreseen by vibration analysis is exceeded by the positive effect due to the impact on a liquid surface. Tests carried out with an optimal load resistance shows that the energy collected by the prototype harvester was larger than most of the values reported in the scientific literature that refers to simple harvesters without a spoon.

Further studies are planned to analyze and improve the performance of the novel harvester. A new series of laboratory tests will be carried out, varying the falling height of the drop and the depth and size of the spoon with the aim of optimizing the water layer. In actual working conditions, the spoon could be equipped with a drainage hole to guarantee the optimal level of water. Experimental tests with actual persistent rain are foreseen in order to study the effect of different impact angles and wind. A raindrop harvester tuned to a certain impact frequency, which corresponds to a specific rain type, could be developed.

## Figures and Tables

**Table 1:** Effect of rigid added mass on the open-circuit voltage.

Test Case	Mean Peak-to-Peak Voltage	Standard Deviation
Harvester with full spoon	3.22 V	0.68 V
Harvester with dry spoon plus 3.0 g added mass	0.65 V	0.15 V
Harvester with dry spoon plus 6.3 g added mass	0.41 V	0.18 V

**Table 2:** Literature values of harvested energy.

Authors	Type of Harvester	Material	Drop Diameter (mm)	Drop Speed (m/s)	Load Resistance (MΩ)	Harvested Energy (μJ)
Guigon et al. [2]	bridge	PVDF	3	4.5	50	0.147
Iljas and Swingler [27]	cantilever	PVDF	4	2	2.5	0.085

## Appendix A

**Table A1:** Substrate materials properties.

Material	Elastic Modulus E [GPa]	Poisson’s Ratio *ν*	Density ρ[kgm3]
Polyester	3.65	0.48	1380
Copper	110	0.34	1300
Steel AISI 304	193	0.29	8000
Polyimide	4.1	0.34	1410

**Table A2:** PZT 5H properties.

Property	Symbol	Value	Unit
Relative permittivity	$\varepsilon_{33}^{T}$	3800	
Piezoelectric constant	$d_{31}$	$-320\;\times \;10^{-12}$	[C/N]
Piezoelectric constant	$d_{33}$	$650\;\times \;10^{-12}$	[C/N]
Young’s modulus	$Y_{11}^{E}$	$6.3\;\times \;10^{10}$	[Pa]
Young’s modulus	$Y_{33}^{E}$	$5.0\;\times \;10^{10}$	[Pa]
Density	ρ	7800	[Kgm3]

**Table A3:** Dimensional properties of the harvester.

Geometric Property	Symbol	Value [mm]
Cantilever Length	L	42.9
Cantilever width	b	23.3
Cantilever thickness	h	0.41
PZT 5H thickness	hPZT	0.15
Steel thickness	hSt	0.15
Copper thickness	hCu	0.03
Polyimide thickness	hPm	0.03
Polyester thickness	hPs	0.05
Lateral edge	Le	1.25
Tip edge	Te	1.80
